# Analysis of human clinical and environmental *Leptospira* to elucidate the eco-epidemiology of leptospirosis in Yaeyama, subtropical Japan

**DOI:** 10.1371/journal.pntd.0010234

**Published:** 2022-03-31

**Authors:** Yukuto Sato, Idam Hermawan, Tetsuya Kakita, Sho Okano, Hideyuki Imai, Hiroto Nagai, Ryosuke Kimura, Tetsu Yamashiro, Tadashi Kajita, Claudia Toma

**Affiliations:** 1 Research Laboratory Center, Faculty of Medicine, University of the Ryukyus, Nishihara, Okinawa, Japan; 2 Center for Strategic Research Project, Organization for Research Promotion, University of the Ryukyus, Nishihara, Okinawa, Japan; 3 Department of Bacteriology, Graduate School of Medicine, University of the Ryukyus, Nishihara, Okinawa, Japan; 4 Department of Biological Sciences, Okinawa Prefectural Institute of Health and Environment, Uruma-shi, Okinawa, Japan; 5 Department of Chemistry, Biology and Marine Science; Faculty of Science, University of the Ryukyus, Nishihara, Japan; 6 Department of Human Biology and Anatomy, Graduate School of Medicine, University of the Ryukyus, Nishihara, Okinawa, Japan; 7 Iriomote Station, Tropical Biosphere Research Center, University of the Ryukyus, Taketomi, Okinawa, Japan; 8 United Graduate School of Agricultural Science, Kagoshima University, Kagoshima, Japan; Tangen Biosciences, UNITED STATES

## Abstract

**Background:**

Leptospirosis, a zoonosis caused by species in the spirochete genus *Leptospira*, is endemic to the Yaeyama region in Okinawa, subtropical Japan. Species of the P1 subclade “virulent” group, within the genus *Leptospira*, are the main etiological agents of leptospirosis in Okinawa. However, their environmental persistence is poorly understood. This study used a combination of bacterial isolation and environmental DNA (eDNA) metabarcoding methods to understand the eco-epidemiology of leptospirosis in this endemic region.

**Findings:**

Polymerase chain reaction (PCR) characterized twelve human clinical *L*. *interrogans* isolates belonging to the P1 subclade “virulent” subgroup and 11 environmental soil isolates of the P1subclade “low virulent” subgroup (genetically related to *L*. *kmetyi*, *n* = 1; *L*. *alstonii*, *n* = 4; *L*. *barantonii*, *n* = 6) from the Yaeyama region targeting four virulence-related genes (*lipL32*, *ligA*, *ligB* and *lpxD1)*. Clinical isolates were PCR positive for at least three targeted genes, while all environmental isolates were positive only for *lipL32*. Analysis of infected renal epithelial cells with selected clinical and environmental strains, revealed the disassembly of cell-cell junctions for the Hebdomadis clinical strain serogroup. Comparison of leptospiral eDNA during winter and summer identified operational taxonomic units corresponding to the species isolated from soil samples (*L*. *kmetyi* and *L*. *barantonii*) and additional P2 subclade species (*L*. *licerasiae*, *L*. *wolffii*-related, among others) that were not detected by soil cultivation. Total *Leptospira* read counts were higher in summer than in winter and the analysis of leptospiral/animal eDNA relationship suggested *Rattus spp*. as a potential reservoir animal.

**Conclusion:**

Our study demonstrated high environmental *Leptospira* diversity in the Yaeyama region, particularly during summer, when most of the leptospirosis cases are reported. In addition, several *Leptospira* species with pathogenic potential were identified that have not yet been reported in Yaeyama; however, the environmental persistence of P1 subclade species previously isolated from human clinical cases in this region was absent, suggesting the need of further methodology development and surveillance.

## Author summary

Leptospirosis is a widespread bacterial zoonosis and one of the most important acute febrile infectious disease in tropical and subtropical regions, which is difficult to differentiate from other infectious diseases common in these regions. Leptospirosis is endemic to Okinawa prefecture, the southernmost prefecture of Japan, where the infection occurs mainly after recreational activities in rivers in the northern part of Okinawa Main Island and the Yaeyama region. This study combined several methods such as bacterial isolation from soil and environmental DNA metabarcoding from river water samples to understand the persistence of *Leptospira* outside the human host, leptospiral diversity in the environment, and their potential reservoir animals in the wild environment of the Yaeyama region. Although this study didn’t confirm the environmental persistence of *Leptospira* species previously isolated from clinical cases, several newly reported *Leptospira* species with pathogenic potential from the Yaeyama region suggested the need for continual surveillance to improve leptospirosis control and prevention in this region.

## Introduction

The genus *Leptospira* comprises 64 species divided into four subclades, called P1, P2, S1, and S2. Subclade P1 can be further split into “virulent” (P1-1 and P1-2 subgroups) and “low virulent” (P1-3, -4, and -5 subgroups) pathogens based on patient outcomes and virulence in animal models [1,2]. Species of the P1 subclade “virulent” subgroups, *Leptospira interrogans*, *L*. *kirschneri*, and *L*. *borgpetersenii*, are the main etiological agents of leptospirosis in Okinawa [3]. Leptospirosis is a bacterial zoonosis with many reservoir hosts, including wild and domestic animals [4]. Pathogenic leptospires colonize the renal tubules of infected reservoir animals, and bacteria are excreted in their urine, contaminating the environment (soil and surface water sources) [5,6]. Humans are exposed to pathogenic leptospires either through direct contact with the urine of infected animals or indirectly through contaminated environmental sources during outdoor activities [7]. Pathogenic *Leptospira* can evade macrophage-mediated killing after penetrating the skin and translocate across the endothelial barrier of the blood vessels to disseminate hematogenously and disassemble the cell-cell junctions to reach the target organs, such as the kidney, lungs, and liver [4,8,9]. Several outer membrane proteins, such as the proteins containing bacterial immunoglobulin-like domains (LigA and LigB), occur exclusively in pathogenic leptospires and are considered virulence factors [4,10]. Additional factors (including LpxD and Lvr) also play important roles in temperature adaptation, modulating outer membrane integrity, and global gene regulation for bacterial adaptation from the environment, such as soil and water, to the animal host [11,12].

The Yaeyama region consists of two major islands, Ishigaki and Iriomote, and other smaller islands in Okinawa, the only subtropical prefecture in Japan. Part of this area was recently listed in UNESCO World Heritage as “Amami-Oshima Island, Tokunoshima Island, Northern part of Okinawa Island, and Iriomote Island”. Due to the subtropical climate, the region is characterized by a dense forest with many waterfalls. The flora and fauna diversity is high and unique, making it a major touristic destination in Japan. Several outdoor attractions such as trekking and water-related recreational activities, including swimming and canyoning, mainly occur during the summer holidays. Tropical diseases, such as malaria and leptospirosis, were historically endemic to the Yaeyama region. Although malaria has been eradicated [13], leptospirosis is still endemic to this region [14]. In Japan, leptospirosis is a notifiable disease since 2003 under the Infectious Diseases Control Low and requires immediate notification of all the diagnosed cases [15]. However, laboratory confirmation is still difficult, and therefore, the number of actual cases is underestimated in Japan, as in other parts of the world [16]. According to the National Epidemiological Surveillance of Infectious Diseases, the annual number of cases in Japan ranges from 16 to 76. More than half of the cases were reported from Okinawa prefecture, mainly in the northern part of mainland Okinawa and the Yaeyama region [14]. In the Yaeyama region, leptospirosis cases have been reported in tourists and recreational guides. Thus, in this region, it is considered a recreational and an occupational infectious disease affecting public health [7].

Although leptospirosis outbreaks have been reported after heavy rain, additional environmental and behavioral factors increase the risk of infection [17]. Bierque *et al*. reported that *L*. *interrogans* retains its direct virulence after long starvation in water [18]. Pathogenic *Leptospira* species are increansingly being isolated from environmental soils around the world [19,20]. Since the survival capacity of leptospires outside a host is determined by the ecological niche and the life cycle of the bacteria is determined by the characteristics of the reservoir animals in each region, different approaches have been used to understand the *Leptospira* life cycle in agricultural, rural, urban, or wildlife environments [21–24]. The culture method developed by Chakraborty *et al*. combining antibiotics to inhibit other soil bacteria [25] allows the isolation of environmental leptospires for their genotypic and phenotypic characterization and has contributed significantly to our understanding of the genus [1,26]. However, the pathogenicity of environmental isolates was not observed in animal infection models; thus, the pathogenicity of some P1 subclade species remains unknown [1]. On the other hand, “virulent” P1 strains are rarely isolated from the environment with the current cultivation protocol. Therefore, molecular methods such as direct detection of leptospiral by qPCR [27] and environmental DNA (eDNA) metabarcoding have been successfully used to increase the detection sensitivity of difficult-to-cultivate leptospiral species and to understand the relationship between *Leptospira* and environmental microbiota and animal reservoirs [28,29].

The National Epidemiological Surveillance System has collected epidemiological data on human leptospirosis since 2003, and leptospirosis cases are recorded almost every year [15]. Moreover, studies on leptospiral reservoir animals in the Okinawa prefecture have shown that the wild boar (S*us scrofa riukiuanus*) is a significant reservoir [30]. However, no study has investigated environmental leptospires in the Yaeyama region. Because of the diversity of animal species and the favorable climate for leptospiral survival outside a host in this region, it is important to understand the *Leptospira* spp. life-cycle in this wild environment to better control and prevent leptospirosis from a One health point of view [31]. This study combined bacterial isolation and eDNA metabarcoding methods to characterize the eco-epidemiology of leptospirosis in the endemic subtropical Yaeyama region of Japan.

## Methods

### Human *Leptospira* clinical isolates

The Okinawa Prefectural Institute of Health and Environment routinely receives specimens for the laboratory confirmation of clinically suspected leptospirosis cases reported in the Yaeyama region. In Japan, leptospirosis is a notifiable disease, and its laboratory confirmation requires at least one of the following criteria: 1) flagellar *flaB* gene positivity by polymerase chain reaction (PCR), 2) culture isolation of *Leptospira* spp., or 3) serological diagnosis by the microscopic agglutination test (MAT).

Human *Leptospira* isolates were obtained between 2007 and 2017 by inoculating two drops of fresh blood into liquid Korthof’s medium (Denka Seiken, Tokyo, Japan) and culturing at 30°C. The serogroups of the isolates were determined by the MAT using 13 antisera for serogroups Australis (serovar Australis), Autumnalis (serovars Autumnalis and Rachmati), Ballum (serovar Castellonis), Bataviae (serovar Bataviae), Canicola (serovar Canicola), Grippotyphosa (serovar Grippotyphosa), Hebdomadis (serovar Hebdomadis) Icterohaemorrhagiae (serovar Icterohaemorrhagiae), Javanica (serovar Javanica), Pyrogenes (serovar Pyrogenes), Pomona (serovar Pomona), and Sejroe (serovar Hardjo).

### Environmental soil and river water sampling

**Fig 1:**
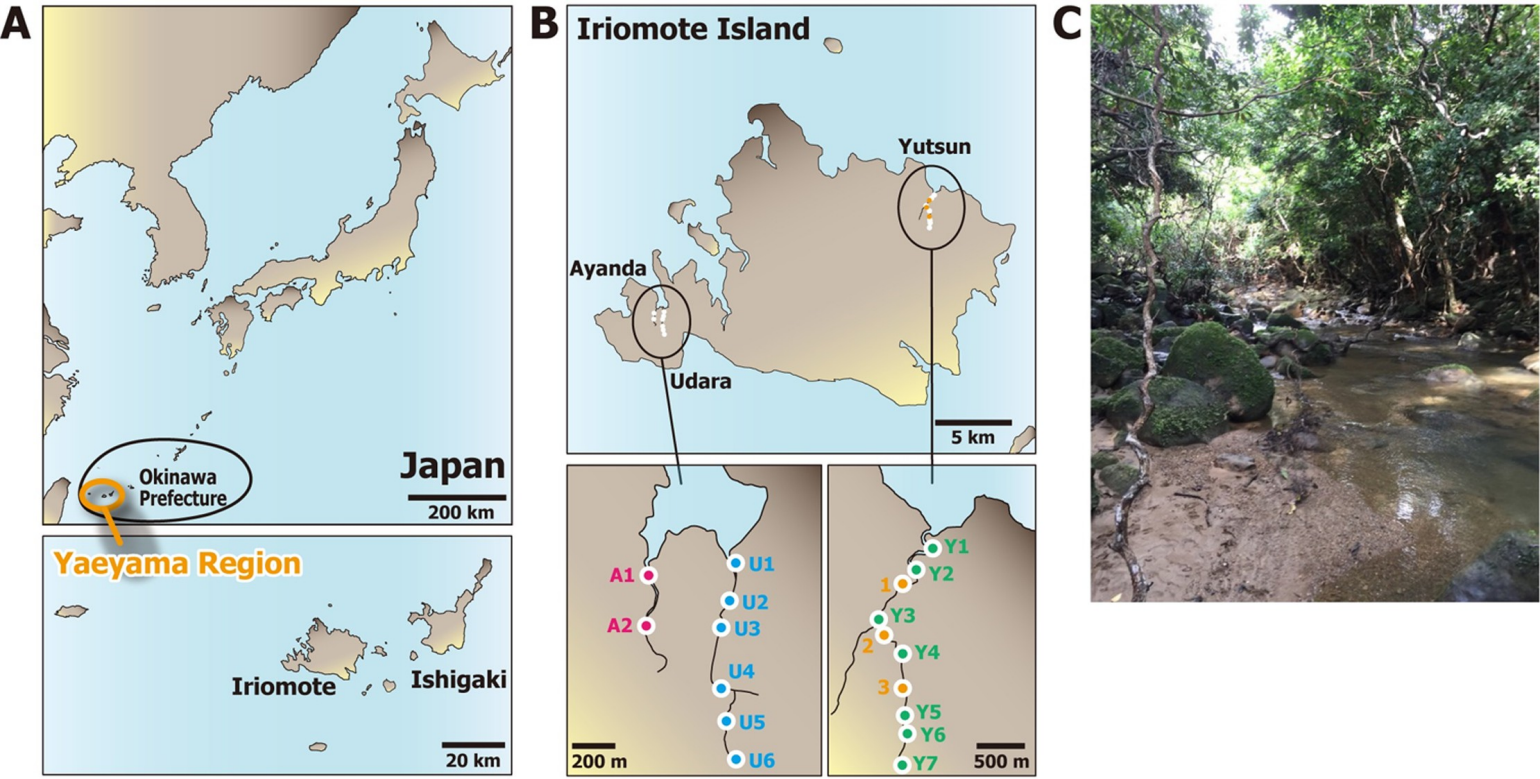
Yaeyama region and locations of soil and river water sampling sites. (A) Locations of Okinawa prefecture and Yaeyama region are shown in the upper left panel, and the Iriomote and Ishigaki Islands are shown in the lower-left panel. The ranges of Okinawa prefecture and Yaeyama region are approximate. (B) Sampling locations alongside of the Yutsun River or Udara and Ayanda Rivers on the east and west sides of the Iriomote Island, respectively. Soil sampling sites are indicated as 1 to 3 (orange circles), and river water sampling sites are as Y1 to Y7 (green circles), U1 to U6 (blue circles), and A1 and A2 (red circles) for Yutsun, Udara, and Ayanda, respectively. The geographical map was drawn by the author using Adobe Illustrator 2021 v25.2 on the basis of map images provided by Geospatial Information Authority of Japan (https://maps.gsi.go.jp) under CC BY 4.0 compatible license. (C) Picture of the representative trekking course along the Yutsun River, soil sampling site 2 (photo credit: Claudia Toma).

Surface soil samples (no deeper than 5 cm) were collected at three sites along the shores of the Yutsun River on Iriomote Island, Yaeyama, where cases of leptospirosis have been reported. On September 24, 2018, eight samples were collected from each sampling site (Fig 1B, orange-colored sites 1 to 3:site 1, YS-1–YS-8; site 2, YS-11–YS-18; and site 3, YS-21–YS-28). Approximately 5 g of soil was placed into 15 mL tubes and kept at room temperature during transportation to the University of the Ryukyus and the samples were processed upon arrival to the laboratory. Environmental *Leptospira* strains were isolated as previously reported, with slight modifications [19,26]. Briefly, 5 mL of 20 mM HEPES buffer (pH 7.4) was added, mixed, and the tubes were incubated for 1 h at 30°C to allow soil decantation. Two mL of the supernatant were transferred to a new tube, followed by the addition of 2.5 mL of 2 x -concentrated Ellinghausen-McCullough-Johnson-Harris (EMJH) broth [Difco Leptospira Medium Base EMJH supplemented with Difco Leptospira Enrichment EMJH (Becton, Dickinson and Company, Franklin Lakes, NJ)], and 0.5 mL of 10 x STAFF (sulfamethoxazole, 400 μg/mL; trimethoprim, 200 μg/mL; amphotericin B, 50 μg/mL; fosfomycin, 4 mg/mL; 5-fluorouracil, 1 mg/mL) [25]. The tubes were cultured at 30°C and observed weekly by a dark-field microscope for one month. Spirochete-positive cultures were filtered with a 0.2 μm pore-size membrane filter, and filtrates (0.5 mL) were added to a new tube containing EMJH broth and further cultured at 30°C for ~10 days until the growth reached the mid-exponential phase. This step was repeated if contaminating bacteria other than spirochetes were observed using a dark-field microscope. *Leptospira* strains were then isolated from LVW agar plates [32]. Agar plates were incubated for 2 days in 5% CO_2_ at 30°C followed by incubation at 30°C in the air for 28 days. Each day, agar plates were observed with the naked eye, and visible colonies were picked up and cultured in 5 mL EMJH broth for DNA extraction.

River water samples were collected from three rivers in Iriomote Island (Fig 1B). Mangroves cover the river mouths of these rivers. Yutsun river is located in a leptospirosis-endemic area on the east side of the island. In contrast, no leptospirosis cases have been reported from the Udara and Ayanda rivers located on the west side (according to the leptospirosis case reports by the Yaeyama Region Public Health Center). Surface water was sampled at seven and six sites of the Yutsun and Udara rivers, respectively, at the edge of the rivers, where the river flow was weaker (Fig 1B). Surface water was taken from a boat at two sites of the Ayanda River. For the Yutsun and Udara rivers, sampling sites covered from head to mouth of the rivers. For the Ayanda river, samples were collected only from the mangrove area at the river mouth.

Environmental water (0.6 L to 1.0 L) was collected at each sampling site by filtering through a Sterivex unit with a 0.45 μm pore size membrane (Merck Millipore, Milan, Italy) attached to a 50 mL disposable syringe SS-50LZ (Terumo, Tokyo, Japan). This filtration unit have been tested to efficiently capture leptospiral cells in our previous study [29]. After filtration, the Sterivex unit was preserved by filling with 2 mL of the DNAiso Reagent (Takara) and tightly sealed with a polypropylene Luer-fitting cap (ISIS, Osaka, Japan). These units were preserved at room temperature during the transfer to a laboratory at the University of the Ryukyus on Okinawa Island for several days and kept at −30°C freezing temperature at the University until DNA extraction. Water samples were taken on January 10, 11 (winter) and July 10, 13 (summer) in 2020. The data on rainfall amount (mm) and temperature (°C) of the Iriomote Island during 2020 was from the website of the Japan Meteorological Agency website (https://www.data.jma.go.jp/obd/stats/etrn/index.php) retrieved on July 5, 2021.

### DNA extraction

DNA from human and soil isolates was extracted from 0.5 ml of EMJH-cultures of single colonies with the DNeasy Blood and Tissue Kit (Qiagen, Hilden, Germany) according to the manufacturer’s protocol and used as a template for PCR.

Environmental DNA was extracted from preserved Sterivex filters using the DNeasy PowerWater Sterivex Kit (Qiagen) according to a standard protocol with a slight modification as follows. Before DNA extraction, the Sterivex units were placed at room temperature to dissolve the DNAiso reagent, which was subsequently removed from the units using a 10 mL disposable syringe (Becton, Dickinson and Company). After adding warmed MBL solution, the filter units were further incubated at 65°C on a heat block for 10 min to efficiently lyse the cells and proteins in the filtrated residues. In the final DNA elution, an ultra-clean 25 μL of microbial DNA-free water (Qiagen) was used to avoid the unexpected contamination from common reagents, obtaining a total of 50 μL eDNA solution from each filter unit through an elution step twice. The eDNA was stored at −30°C after verification of its concentration and quality (OD_260/280_ >1.80) using a Nanodrop 2000c Spectrophotometer (Thermo Fisher Scientific).

### PCR amplification and sequencing of the 16S rRNA gene for determination of *Leptospira* species of human and soil isolates

Human and soil isolates DNA was used as a template for the PCR targeting the leptospiral 16S rRNA gene using primer pairs 8UA and 1485R (universal for all *Leptospira* species) (Table 1). PCR products were purified using spin columns (LaboPass PCR, Hokkaido System Science, Japan) and subjected to shotgun DNA sequencing using the Illumina MiSeq platform (Illumina, San Diego, CA, USA) with the Nextera XT DNA Library Prep Kit and the Index Kit v2 (Illumina) according to the manufacturer’s protocol with the following modifications. All reaction volumes were scaled down to a quarter, and the DNA fragmentation/tagmentation step was carried out for 25 min. The Nextera PCR mix was replaced with the same volume of HiFi HotStart ReadyMix (Kapa Biosystems, Woburn, MA, USA) for efficient library amplification with an increase of 18 PCR cycles. The obtained tag-indexed shotgun libraries from each sample were pooled in equal amounts, and the 400–700 base pairs (bp)-sized fragments were purified using the 1.0% L03 agarose gel (Takara, Shiga, Japan), the MinElute Gel Extraction Kit (Qiagen), and the AMPure XP magnetic beads (Beckman Coulter, High Wycombe, UK) according to standard purification protocols. The sequencing library obtained was quantified using Qubit 3.0, using the dsDNA HS Assay Kit (Thermo Fisher Scientific, Waltham, USA). The 20 pM library was sequenced using the MiSeq Nano flow cell and v2 250-bp paired-end sequencing chemistry (Illumina).

**Table 1:** List of primers used for PCR analysis or environmental DNA metabarcoding sequencing by Illumina MiSeq platform.

Primer names	Target gene	Sequence (5’–3’)^1^^,^^2^	References
8UA	Leptospiral 16S rRNA	AGAGTTTGATCMTGGCTCAG	[19]
1485R	Leptospiral 16S rRNA	TACGGYTACCTTGTTACGACTT	[19]
LipL32-45F	*lipL32*	AAGCATTACCGCTTGTGGTG	[36]
LipL32-Rb	*lipL32*	GAACTCCCATTTCAGCGAT	[36]
L-*flaB-*F1	*flaB*	CTCACCGTTCTCTAAAGTTCAAC	[37]
L-*flaB-*R1	*flaB*	TGAATTCGGTTTCATATTTGCC	[37]
LigA-F	*ligA*	GTTAATGCACTCTCGAGGAGG	This study
LigA-R	*ligA*	ATCTCCAAATGCAAGAGCCG	This study
LigB-F	*ligB*	ACGGAACTTTTCCGGAATCT	[10]
LigB-R	*ligB*	AACCGGGATACCTCCATCTC	[10]
lpxD1-F	*lpxD1*	TACGTATTTATTGCCGGTGG	This study
lpxD1-R	*lpxD1*	CCAAAATAAGCAACTTTCTC	This study
Lepat 1	Leptospiral 16S rRNA	ACACTCTTTCCCTACACGACGCTCTT CCGATCTNNNNNNGAGTCTGGGATAACTTT	[22]
Lepat 2	Leptospiral 16S rRNA	GTGACTGGAGTTCAGACGTGTGCTC TTCCGATCTNNTCACATCGYTGCTTATTTT	[22]
16S rRNA V4 F	Bacterial 16S rRNA	ACACTCTTTCCCTACACGACGCTCTTCCGA TCTNNNNNNGTGCCAGCMGCCGCGGTAA	[38]
16S rRNA V4 R	Bacterial 16S rRNA	GTGACTGGAGTTCAGACGTGTGCTCTTCC GATCTNNNNNNGGACTACHVGGGTWTCTAAT	[38]
MiFish-U-F	Vertebrate mitochondrial 12S rRNA	ACACTCTTTCCCTACACGACGCTCTTCCG ATCTNNNNNNGTCGGTAAAACTCGTGCCAGC	[35]
MiFish-U-R	Vertebrate mitochondrial 12S rRNA	GTGACTGGAGTTCAGACGTGTGCTCTTCCG ATCTNNNNNNCATAGTGGGGTATCTAATCCCAGTTTG	[35]

^1^ Positions with mixed bases are designated by their IUB codes:R = A/G; Y = C/T; K = G/T; M = A/C; S = G/C; W = A/T.

^2^ Target-specific nucleotide regions are denoted by the underline.

The sequence data were quality filtered, and *de novo* assembled to estimate the 16S rRNA gene sequences and the molecular phylogeny with the reference *Leptospira* sequences. The low-quality (Phred score <10) 3’-tails of each read were trimmed using the program DynamicTrim of the SolexaQA software package [33]. The Nextera adaptor sequences that repeatedly appeared or remained on the reverse complement end of the reads were removed by Cutadapt [34] with a threshold of ≤10% base mismatches. The shorter <80 bp reads were removed using a custom Perl script (available at http://dx.doi.org/10.5061/dryad.54v2q; Miya *et al*. [35]; usable for the present analysis by changing the cutoff value into 80 bp using ordinal text editor), and these quality-filtered sequences were assembled using the CLC Genomics Workbench version 9.5.2 (Qiagen) with default parameters. The successfully assembled contigs (ca. 1,500–1,600 bp) with a minimum sequencing depth ≥4 and consensus nucleotide bases determined under a majority rule and quality scores (avoiding the use of degenerate bases) were subjected to phylogenetic analysis.

### Phylogenetic analysis

The representative 16S rRNA gene sequences of the genus *Leptospira* were collected based on the phylogenetic trees of Guglielmini *et al*. (2019) [1] and Vincent *et al*. (2019) [2]. These reference sequences and the 16S rRNA sequences determined in the present study were aligned using MAFFT version 7.310 [39] and analyzed by the neighbor-joining (NJ) method [40] based on the Kimura’s two-parameter model of nucleotide substitution [41] using MEGA X version 10.2.5 [42]. The support values for the tree node were estimated using 1,000 replications of bootstrapping analysis. We considered that ≥50% of the bootstrap support value was reliable and indicated it on the nodes of the phylogenetic trees. In the preliminary analysis, the maximum-likelihood (ML) phylogenetic analysis was also conducted for the same data set. The resultant phylogenetic tree, however, was more compatible with the reference leptospiral trees of Guglielmini *et al*. (2019) [1] and Vincent *et al*. (2019) [2] in the NJ method than in ML method, probably because the present data set include relatively higher number of closely related sequences with fewer number of saturated nucleotide sites. Accordingly, we adapted the NJ method for the phylogenetic analyses of the present study.

### Characterization of human and soil *Leptospira* isolates by PCR

Human and soil environmental leptospiral isolates were further characterized by PCR targeting the flagellar *flaB* gene and virulence-related genes, *lipL32*, *ligA*, *ligB* and *lpxD1*, using the primers listed in Table 1. The primers to detect *ligA* and l*pxD1* genes were designed in this study using Primer3 [43,44] based on *L*. *interrogans* serovar Manilae sequences [45], GenBank accession CP011931 (locus tag LIMLP_15405 for *ligA* and locus tag LIMLP_02200 for *lpxD1*). The PCRs were performed using LaboPass G-Taq (Hokkaido System Science) in a total reaction volume of 20 μL for 30 cycles of amplification at an annealing temperature of 55°C.

### PCR amplification of water eDNA and sequencing for metabarcoding analysis

Using a two-step tailed PCR method, we amplified and sequenced partial fragments of the 16S rRNA gene of *Leptospira*, which is more specific than *lipL32* gene when using environmental samples [28], and the mitochondrial (mt) 12S rRNA genes of broad vertebrate animals from the prepared eDNA as previously described [28,29]. The known PCR primers targeting *Leptospira* 16S rRNA [22] and vertebrate mt-12S rRNA [35] were used along with additional MiSeq sequencing priming sites and random hexamer or dimer nucleotides to improve basecall calibration of the MiSeq (Illumina) (Table 1). The typical length of PCR fragments and the final concentration of primers were 330 and 169 bp, and 0.38 and 0.29 μM for leptospiral 16S rRNA and vertebrate mt-12S rRNA, respectively. For the former PCR, the Multiplex PCR Assay Kit version 2 (Takara) was used with 2.0 μL of template eDNA in a total reaction volume of 10.0 μL through 37 cycles of amplification at an annealing temperature of 50°C with additional bacterial universal 16S rRNA V4 primers (Table 1) at the same concentration as the positive control. For the latter PCR, the HiFi HotStart ReadyMix (Kapa Biosystems) was used with 1.5 μL of templates in a total reaction volume of 12.0 μL through 35 amplification cycles with two patterns of annealing temperatures of 60°C and 65°C. The 1st-round PCR products were diluted into RNase-free water (Thermo Fisher Scientific) for 30- and 20-fold in leptospiral 16S rRNA and vertebrate mt-12S rRNA, respectively, and subjected to 2nd-round PCR to add the dual-index tags (D5 and D7 series) and MiSeq flow cell-binding sites (Illumina) using Ex Taq Hot Start Version (Takara) as previously described [29,38].

The tag-indexed 2nd-round PCR products were sequenced using MiSeq (Illumina). The PCR products with unique combinations of dual indices were pooled in equal amounts for semi-quantitative purposes and purified using the 1.0% L03 agarose gel (Takara) and a MinElute Gel Extraction Kit (Qiagen) under a standard protocol. The eluted DNA solution was further purified and concentrated using AMPure XP magnetic beads (Beckman Coulter) with a standard purification protocol. The obtained sequencing library was quantified using the Qubit 3.0 with the dsDNA HS Assay Kit (Thermo Fisher Scientific). The 30 pM sequencing library was subjected to DNA sequencing using the MiSeq Reagent Kit v3 600 or Micro v2 300 Cycles Kit (Illumina) for 301 or 151 bp paired-end sequencing of leptospiral 16S rRNA or vertebrate mt-12S rRNA genes, respectively. The volume molarity of the libraries was estimated based on the average molecular weight of a DNA nucleotide (660 g per 1 bp), DNA concentration, and the mean length (bp) of the second-round PCR products of the library: 526, 454, and 365 bp in leptospiral 16S rRNA, bacterial 16S rRNA V4, and vertebrate mt-12S rRNA genes, respectively.

### Metabarcoding data analysis of *Leptospira* and potential host animals

Sequence data generated by the MiSeq underwent primary processing based on data quality. The low-quality 3-tail nucleotides of each sequence with an error rate >10^−1^ were removed using the program DynamicTrim of the SolexaQA software package [33]. The tail-trimmed paired-end sequences were then connected using FLASH software [46] and processed by custom Perl scripts to exclude sequences containing basecall failures (N bases; available at http://dx.doi.org/10.5061/dryad.54v2q; Miya *et al*. [35]) and showing atypical length compared to the expected sizes of PCR products described above. Primer sequences were removed using TagCleaner [47], with a maximum five-base mismatch. Sequences without primers at either end were removed. The redundant identical sequences within each sample were merged into a dereplicated sequence while keeping its count information using UCLUST (derep_fulllength command) [48]. Finally, the singleton sequences in each sample lacking reproducibility were aligned with effective ≥2 counts sequences at ≥99% sequence similarity to absorb sequencing error variations. The number of aligned singletons was added to the count information of the matched effective sequence, and the unmapped singletons were discarded at this step.

These quality-filtered effective sequences were analyzed to estimate their taxonomic origin based on sequence similarity analysis to known reference sequences by using the National Center for Biotechnology Information (NCBI) BLAST plus program [49]. The NCBI nucleotide (nt) [50] was used to analyze the leptospiral 16S rRNA gene as a reference database. The MitoFish [51,52] and the NCBI nt were used to analyze the vertebrate mt-12S rRNA gene. For the leptospiral 16S rRNA, the Blastn analysis was performed at moderate sequence similarity and an *e*-value threshold of 85% and 10^−3^, respectively, to avoid false negatives (type II errors) in this preliminary sequence annotation. The provisional annotations according to the BLAST top-hit results were confirmed or corrected based on the molecular phylogenetic analysis with known representative sequences as described in the **“Phylogenetic analysis”** section. The sequence counts of each species from the respective PCR replicates were summed for each sample.

For the vertebrate mt-12S rRNA gene, the Blastn-based species annotation was performed at the sequence similarity and *e*-value thresholds of 90% and 10^−5^, respectively, as the database completeness of vertebrate mtDNA was generally higher than that of bacteria or *Leptospira*. The sequence counts of the species from two separate PCRs with different annealing temperatures (60°C and 65°C) were summed for each sample.

### Statistical analysis

Significance of the difference in average values was examined by Welch’s *t* test or one way ANOVA (analysis of variance) based on mean and standard error or variance scores. These tests were applied for the analysis of the amount of rainfall (mm), concentration and quality values of the extracted eDNA, detected numbers of the leptospiral sequences, and Shannon index of alfa-diversity of the detected leptospiral OTUs. The possible association between the sequence numbers between leptospiral and vertebrate species was tested based on a Pearson’s product-moment correlation coefficient (*r*). It’s false discovery rate (FDR) was estimated and evaluated as *q* value based on the Benjamini-Hochberg method. The correlation between leptospiral and vertebrate sequence detection was also examined by converting to presence/absence data using two-tailed Fisher’s 2 x 2 exact test. The parameters estimation was performed using the software PAST version 4.06b [53].

### Cell culture and infection

RPTEC/TERT1 (ATCC CRL-4031) cells were grown in Dulbecco’s modified Eagle medium/nutrient mixture F-12 Ham (DMEM/F-12 Ham; Sigma-Aldrich) supplemented with 5 pM triiodothyronine, 10 ng/mL recombinant human epidermal growth factor, 3.5 μg/mL ascorbic acid, 5 μg/mL transferrin, 5 μg/mL insulin, 8.65 ng/mL sodium selenite and 100 μg/mL G418. The cells were seeded at a density of 1.3 × 10^6^ cells per well in polyethylene terephthalate hanging cell culture inserts, with a pore size of 1 μm (Millipore). Cells were maintained in a humidified incubator at 37°C and 5% CO_2_ for 7 to 10 days after reaching confluence to allow monolayer maturation, and the medium was exchanged every 2 days [9].

The cells were infected with leptospires at a multiplicity of infection (MOI) of 100 from the basolateral side containing supplement-free DMEM/F-12 Ham medium and the transepithelial electrical resistance (TEER) of the monolayers was measured using a Millicell-ERS cell resistance indicator (Millipore) as previously described [9]. After subtracting of the value of a cell-free filter (blank), the mean TEER value was expressed as Ωcm^2^. The TEER of the cells before infection was designated as the baseline value. The percentage of TEER relative to the baseline value was calculated using the following formula: (TEER of experimental wells/baseline TEER of experimental wells) ×100%.

### Immunostaining of infected cells

To analyze the monolayer integrity at the cellular level, occludin, a membrane protein of the apical junctional complex was immunostained [54]. RPTECs were fixed in cold methanol for 15 min, permeabilized, and blocked with 5% bovine serum albumin (BSA) and 1% Triton X-100 in Tris-buffered saline (TBS; 50 mM Tris and 150 mM NaCl [pH 7.4]) for 15 min. Mouse monoclonal anti-occludin (1:50; sc-133256, Santa Cruz) was diluted in TBS containing 1% BSA and 0.1% Triton X-100, and anti-mouse Alexa 488 (1:100; Jackson ImmunoResearch #715-545-151) was used as a secondary antibody. The cells were counterstained with a 1:100 dilution of TO-PRO-3 (Invitrogen) to label the DNA. The compiled Z-stack images were acquired on a Leica TCS-SPE confocal laser scanning microscope after mounting with SlowFade Diamond Antifade Mountant (Invitrogen) using LEICA LAS AF acquisition software (Leica Microsystems).

## Results

### Molecular characterization of *Leptospira* human clinical isolates

**Fig 2:**
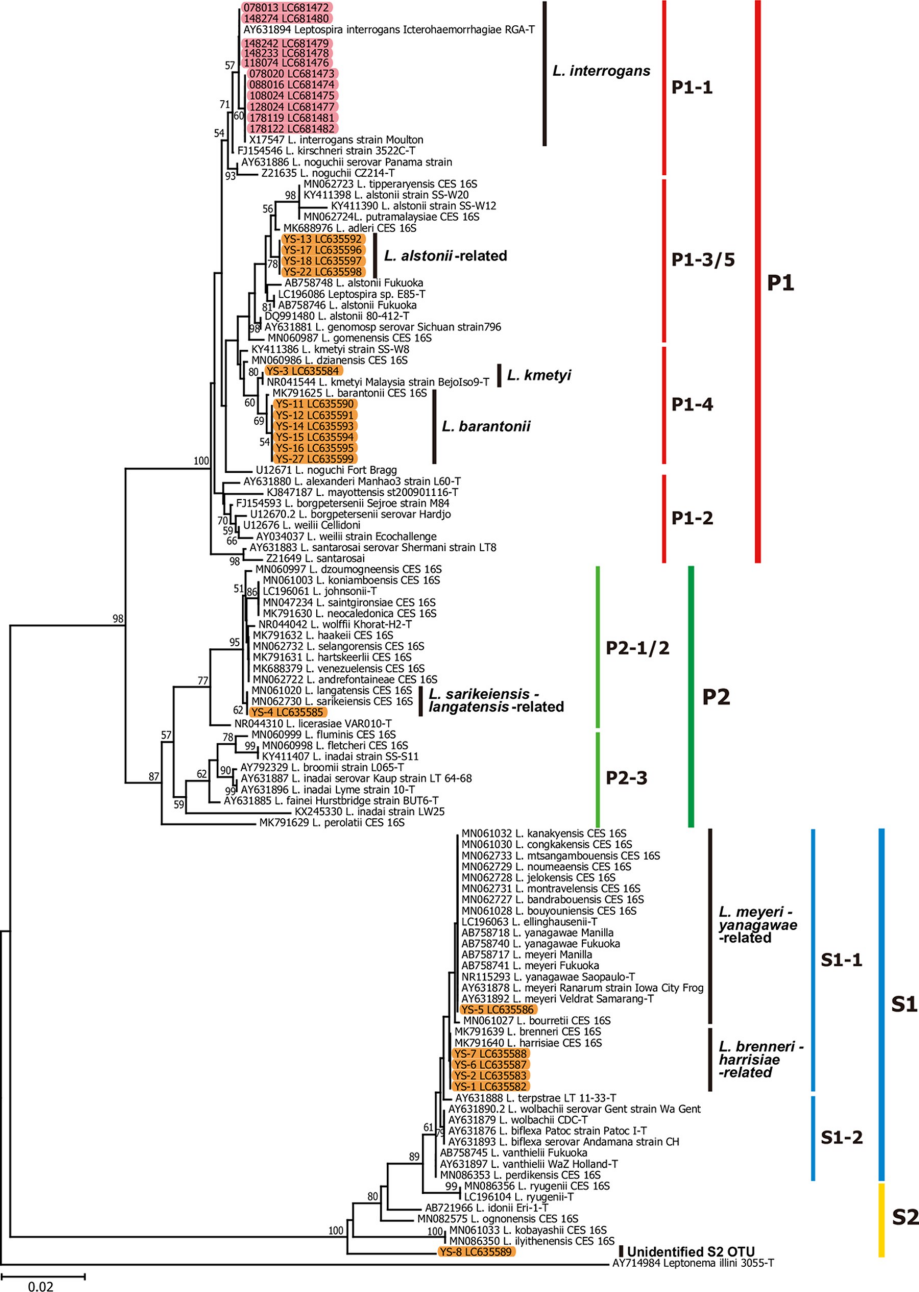
Molecular phylogenetic tree of leptospiral 16S rRNA sequences from human and soil isolates with those of representative *Leptospira* species. Red and orange shading indicate the 16S rRNA genes determined from *Leptospira* cultures from human patients and soil samples of the present study, respectively (ranging from 1,431 to 1,581 bp). The GenBank accession numbers of known 16S rRNA sequences of representative *Leptospira* species and those isolated in this study were shown in the sequence names. In total, 620 nucleotide sites among these 115 sequences were aligned and analyzed by the neighbor-joining method with Kimura’s two parameter model of nucleotide substitution. The 16S rRNA sequence of the human isolate strain 178127 (DDBJ accession number LC681483) was excluded from the phylogenetic analysis because of its shorter sequence length (1,225 bp), while the sequence was estimated as *L*. *interrogans* by the Blast-based analysis with 100% sequence match. Values on the tree nodes denote percentage support for the node estimated from 1,000 bootstrap replications. Subclades (P1, P2, S1, S2) and subgroups annotations P1-1 to -5, P2-1 to -3, S1-1 and -2, and S2 are based on Guglielmini *et al*. (2019) [1].

This study analyzed twelve *Leptospira* human isolates from patients reported between 2007 to 2017 in the Yaeyama region. They belonged to *L*. *interrogans* serogroup Hebdomadis (*n* = 7), serogroup Icterohaemorrhagiae (*n* = 1), and serogroup Grippotyphosa (*n* = 4) (Table 2, Fig 2). After PCR, all isolates were positive, with primers designed to detect *flaB* of P1 pathogenic species (Table 1) and the genes encoding outer membrane proteins *lipL32* and *ligB* (Tables 1 and 2). Interestingly, isolates of the Hebdomadis serogroup were negative for the outer membrane protein gene *ligA* with the PCR primers designed in this study (Table 2). Thus, to further characterize the clinical isolates, we focused on the *lpxD1* gene, described as an important gene for maintaining outer membrane integrity when *Leptospira* is transmitted from the environment to the animal host [11]. All clinical isolates were positive for *lpxD1* (Table 2), which agrees with their pathogenic potential since LpxD1 plays an important role in temperature adaptation and virulence in the host [11].

**Table 2:** Characterization of *Leptospira* human clinical isolates from the Yaeyama region.

Isolate ID	Year	Location	Serogroup	PCR				
				*flaB*	*lipL32*	*ligB*	*ligA*	*lpxD1*
078013	2007	Ishigaki	Icterohaemorrhagiae	+	+	+	+	+
078020	2007	Ishigaki	Hebdomadis	+	+	+	-	+
088016	2008	Iriomote	Hebdomadis	+	+	+	-	+
108024	2010	Iriomote	Hebdomadis	+	+	+	-	+
118074	2011	Iriomote	Grippotyphosa	+	+	+	+	+
128024	2012	Iriomote	Hebdomadis	+	+	+	-	+
148233	2014	Iriomote	Grippotyphosa	+	+	+	+	+
148242	2014	Ishigaki	Grippotyphosa	+	+	+	+	+
148274	2014	Ishigaki	Grippotyphosa	+	+	+	+	+
178119	2017	Iriomote	Hebdomadis	+	+	+	-	+
178122	2017	Iriomote	Hebdomadis	+	+	+	-	+
178127	2017	Iriomote	Hebdomadis	+	+	+	-	+

### Characterization of soil environmental *Leptospira* isolated by culturing

The occurrence of leptospirosis is related to water recreational activities such as canoeing and trekking by tourists and instructors in the Yaeyama region. To contribute to the understanding of environmental leptospires, we characterized isolates obtained along the shores of the Yutsun River on Iriomote Island (Fig 1). A total of 24 soil samples were collected from three sampling sites, and 18 *Leptospira* spp. isolates were recovered by culturing and phylogenetic analyzed (Fig 2). Soil environmental *Leptospira* isolates belonged to the P1 subclade (*n* = 11), P2 subclade (*n* = 1), S1 subclade (*n* = 5) and S2 subclade (*n* = 1). All environmental isolates belonging to the P1 subclade included species of the “low virulence” subgroups (genetically related to *L*. *kmetyi*, *n* = 1; *L*. *barantonii*, *n* = 6; or *L*. *alstonii*, *n* = 4) that have not been reported to date in the Yaeyama region. *Leptospira* species belonging to the “virulent” subgroups associated with clinical cases were not isolated around the Yutsun River. Environmental isolates belonging to the P1 subclade were positive after PCR targeting *flaB* and *lipL32* genes. However, the PCRs for *ligA*, *ligB* and *lpxD1* were negative for all P1 “low virulence” subgroups, showing that these PCR primers are specific for P1 “virulent” subgroups (Table 3).

**Table 3:** Characterization of environmental *Leptospira* isolates.

Isolate ID	Sampling site	Subclade (Subgroup)	Species (genetically related to)	PCR				
				*flaB*	*lipL32*	*ligA*	*ligB*	*lpxD1*
YS-1	1	S1 (S1-1)	*L*. *brenneri*	-	-	-	-	-
YS-2	1	S1 (S1-1)	*L*. *brenneri*	-	-	-	-	-
YS-3	1	P1 (P1-4)	*L*. *kmetyi*	+	+	-	-	-
YS-4	1	P2 (P2-2/1)	*L*. *sarikeinensis*	-	-	-	-	-
YS-5	1	S1 (S1-1)	*L*. *meyeri*	-	-	-	-	-
YS-6	1	S1 (S1-1	*L*. *brenneri*	-	-	-	-	-
YS-7	1	S1 (S1-7)	*L*. *brenneri*	-	-	-	-	-
YS-8	1	S2 (u.i.)*	Unidentified	-	-	-	-	-
YS-11	2	P1 (P1-4)	*L*. *barantonii*	+	+	-	-	-
YS-12	2	P1 (P1-4)	*L*. *barantonii*	+	+	-	-	-
YS-13	2	P1 (P1-3/5)	*L*. *alstonii*	+	+	-	-	-
YS-14	2	P1 (P1-4)	*L*. *barantonii*	+	+	-	-	-
YS-15	2	P1 (P1-4)	*L*. *barantonii*	+	+	-	-	-
YS-16	2	P1 (P1-4)	*L*. *barantonii*	+	+	-	-	-
YS-17	2	P1 (P1-3/5)	*L*. *alstonii*	+	+	-	-	-
YS-18	2	P1 (P1-3/5)	*L*. *alstonii*	+	+	-	-	-
YS-22	3	P1 (P1-3/5)	*L*. *alstonii*	+	+	-	-	-
YS-27	3	P1 (P1-4)	*L*. *barantonii*	+	+	-	-	-

* u.i.: unidentified S2 OTU (see Fig 2)

### Renal proximal tubule epithelial cells (RPTEC) infection by *Leptospira* isolates

**Fig 3:**
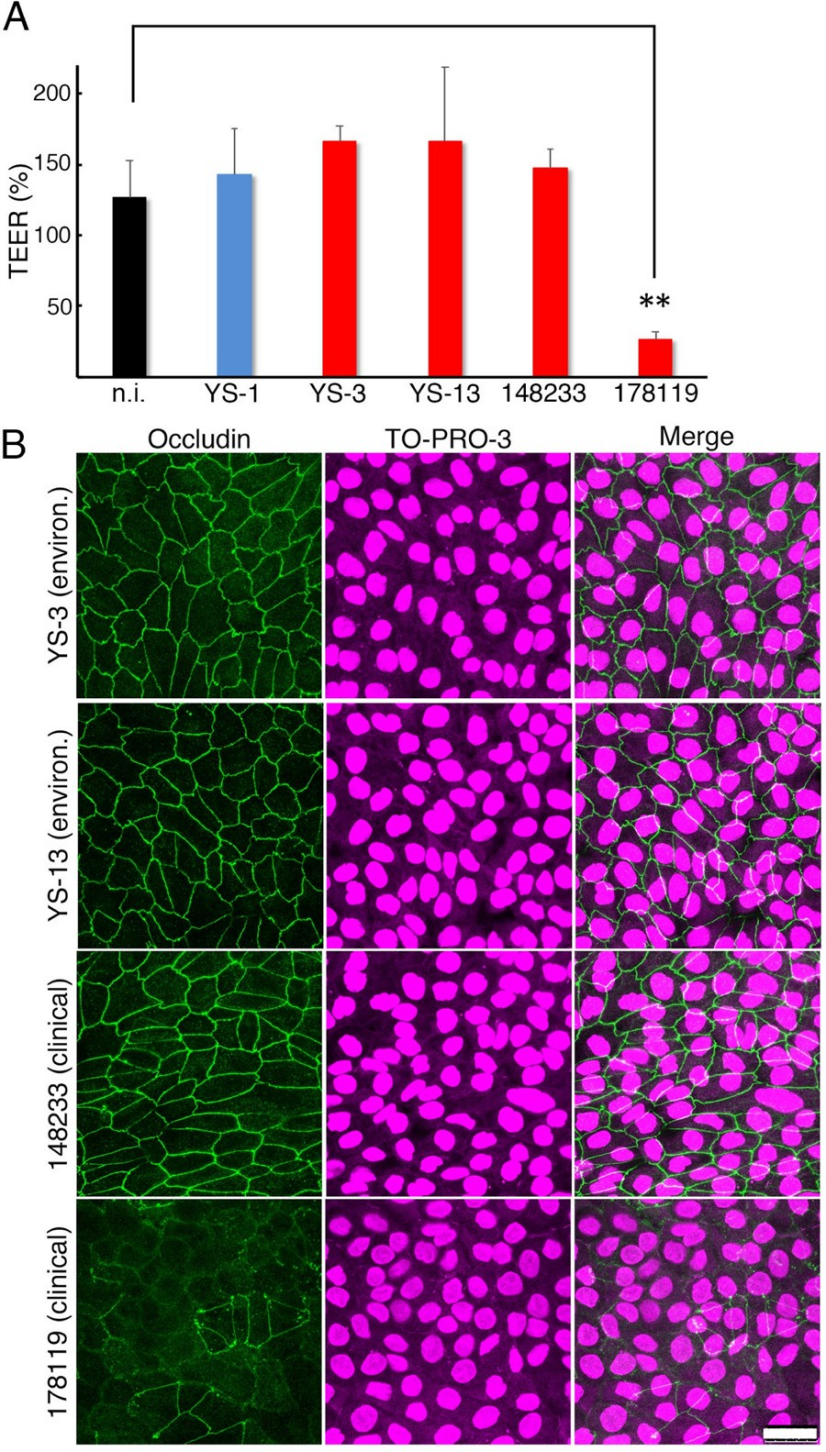
RPTEC infection by environmental and clinical isolates. RPTECs were infected with *Leptospira* species of the subclade S1 or P1 and evaluated 24 h post-infection for their ability to disrupt cell-cell junctions. **(A)** Monolayer integrity was evaluated by measuring TEER. Polarized RPTECs were infected from the basal side with isolates of the subclades S1 (YS-1, blue) or P1 (YS-3, YS-13, 148233 and 178119, red). Non-infected (n.i.) RPTECs (black) were used as controls. The values are the mean ± S.D. of three independent experiments. ** *P* < 0.01 (unpaired two-tailed Student’s *t* test). **(B)** Representative confocal images of infected RPTECs with environmental (YS-3 and YA-13) or clinical (148233 and 178119) isolates. Infected cells were fixed, and 24 h p.i., occludin was stained with an Alexa-Fluor 488-labelled antibody (green), the cell nuclei were counterstained with TO-PRO-3 (magenta). Scale bar 25 μm.

Sebastián *et al*. have reported that pathogenic leptospires can induce cell-cell junction disassembly for bacterial dissemination across epithelial cells [9]. To evaluate whether environmental and clinical Yaeyama isolates can disrupt the cell-cell junctions, we selected representative isolates of S1 and P1 subclades and analyzed their ability to disturb RPTEC monolayer integrity after 24 h post-infection (p.i.) by measuring the transepithelial electrical resistance (TEER) [55]. Environmental isolates (YS-1, YS-3, and YS-13) did not decrease the TEER at 24 h p.i. (Fig 3A) regardless of their genomic classification (S1 or P1 subclade). However, the clinical serovar Hebdomadis strain 178119 showed a significant decrease in the TEER. In the contrast, the TEER did not decrease in the serovar Grippotyphosa strain 148233-infected RPTECs (Fig 3A).

Since a decrease in the TEER in 178119-infected RPTECs suggested the disassembly of the cell-cell junctions, we next investigated the localization of occludin, a transmembrane protein involved in maintaining the cellular barrier [54] at the cellular level by confocal microscopy. As expected, immunofluorescence showed that occludin was displaced from the plasma membrane after infection with 178119 (Fig 3B), but it remained at the plasma membrane after infection with YS-3, YS-13, and 148233. These results suggest that 178119 expresses essential factors that disrupt the cell-cell junctions. However, some clinical isolates, such as 148233, might not disrupt cell-cell junctions or may become avirulent during several *in vitro* passages and/or long-term storage in the laboratory [10]. Environmental isolates may not disassemble the apical junctional complex or may loss this ability due to several passages during the isolation procedure.

### Environmental DNA collection and metabarcoding sequencing of *Leptospira*

To offset the limitations of culturing methods and address a broader view of the eco-epidemiology of leptospirosis in Iriomote Island, we performed an eDNA metabarcoding analysis targeting the genus *Leptospira* [28,29]. The analysis aimed to evaluate and compare the diversity of leptospires across three rivers from the east to the west side of the island and between the summer and winter (Fig 1). Between these seasons, there was no significant difference in the amount of rainfall (mm) during the sampling periods (including two days before sampling, the day before the sampling, and the day of sampling; 0.50 ± 0.50 and 1.00 ± 0.76 mm in summer and winter, respectively; Welch’s *t* test, *d*.*f*. = 5.54, *t* = 0.55, *p* = 0.604).

We obtained highly pure eDNA in general in thirty samples (14, 12, and 4 from Yutsun, Udara, and Ayanda Rivers, respectively) with an average concentration and OD_260/280_ quality values of 1.88 ± 0.22 ng/μL (mean ± S.E.) and 3.21 ± 0.63 (S1 Table). One thousand mL of river water was filtered on most samples (63.3% [19/30]) and at least 600 mL of water was filtered through the remaining sampling until the Sterivex filter was clogged up with suspended matter (on average 918 mL; 900 to 1,000 mL, 600 to 1,000 mL, and 600 to 800 mL in Yutsun, Udara, and Ayanda rivers, respectively; S1 Table). The concentration and quality of the eDNA obtained showed no significant difference among the three rivers (one way ANOVA, *d*.*f*. = 13; *F* = 2.40, *p* = 0.850 in concentration ng/μL and *F* = 2.40, *p* = 0.984 in OD_260/280_ value). On the other hand, the summer samples exhibited significantly higher concentration of the eDNA (2.35 ± 0.40 and 1.40 ± 0.13 ng/μL in summer and winter, respectively; Welch’s *t* test, *d*.*f*. = 16.9, *t* = 2.26, *p* = 0.037), while their quality OD_260/280_ were not significantly different between the seasons (3.56 ± 1.13 and 2.87 ± 0.59 in summer and winter, respectively; Welch’s *t* test, *d*.*f*. = 21.1, *t* = 0.54, *p* = 0.594).

A partial fragment of the pathogenic leptospiral 16S rRNA gene was amplified from each eDNA sample using a pair of “Lepat-1 and -2” primers (Table 1) [22]. The amplification was performed by a multiplex PCR with broad bacterial 16S rRNA gene V4 region using “16S rRNA V4-F and -R” primers (Table 1) [29,38] as positive control amplicons. The PCR was replicated twice for each sample and sequenced independently, producing a total of 13,289,584 pairs of raw sequences with 221,493 ± 3,454 reads per replicate (ranging from 159,350 to 276,690 reads per replicate; DRA012239). After quality-based filtering of the reads, a total of 11,825,523 sequences remained as quality-filtered sequences, with 197,092 ± 3,172 sequences per replicate. Among them, both primer ends of the pathogenic leptospiral 16S rRNA gene were found in 33,012 sequences (550 ± 132 reads per replicate; ranging from 0 to 4,449 per replicate) and those of the broad bacterial 16S rRNA gene V4 region were from 11,792,511 sequences (196,542 ± 3,131 reads per replicate; ranging from 143,105 to 245,140 per replicate).

From the filtered 33,012 sequences of pathogenic leptospiral 16S rRNA gene, we found a total of 27,572 non-singleton (≥2 counts) effective sequences after rescuing singleton reads by re-mapping analysis and by preliminary Blast-based taxonomic annotation (for details, see Methods). The average number of the effective leptospiral sequences was 460 ± 122, ranging from 0 to 3,949 per a replicate. The remaining sequences were singletons in a replicate or sequences without hits or those with a hit to other unknown bacteria in the BLAST database (2,384, 3,046, and 10 sequences, respectively). *Leptospira* total read numbers were higher in summer (mean daily temperature of 29.6°C and ranging from 27.3°C to 32.3°C on July 2020), when the climate conditions are favorable for leptospiral persistence in the environment, compared to winter (mean daily temperature of 19.4°C and ranging from 16.9°C to 22.4°C on January 2020). However, the read number difference was not significant, accordingly the results suggested that there is no difference between winter and summer.

**Fig 4:**
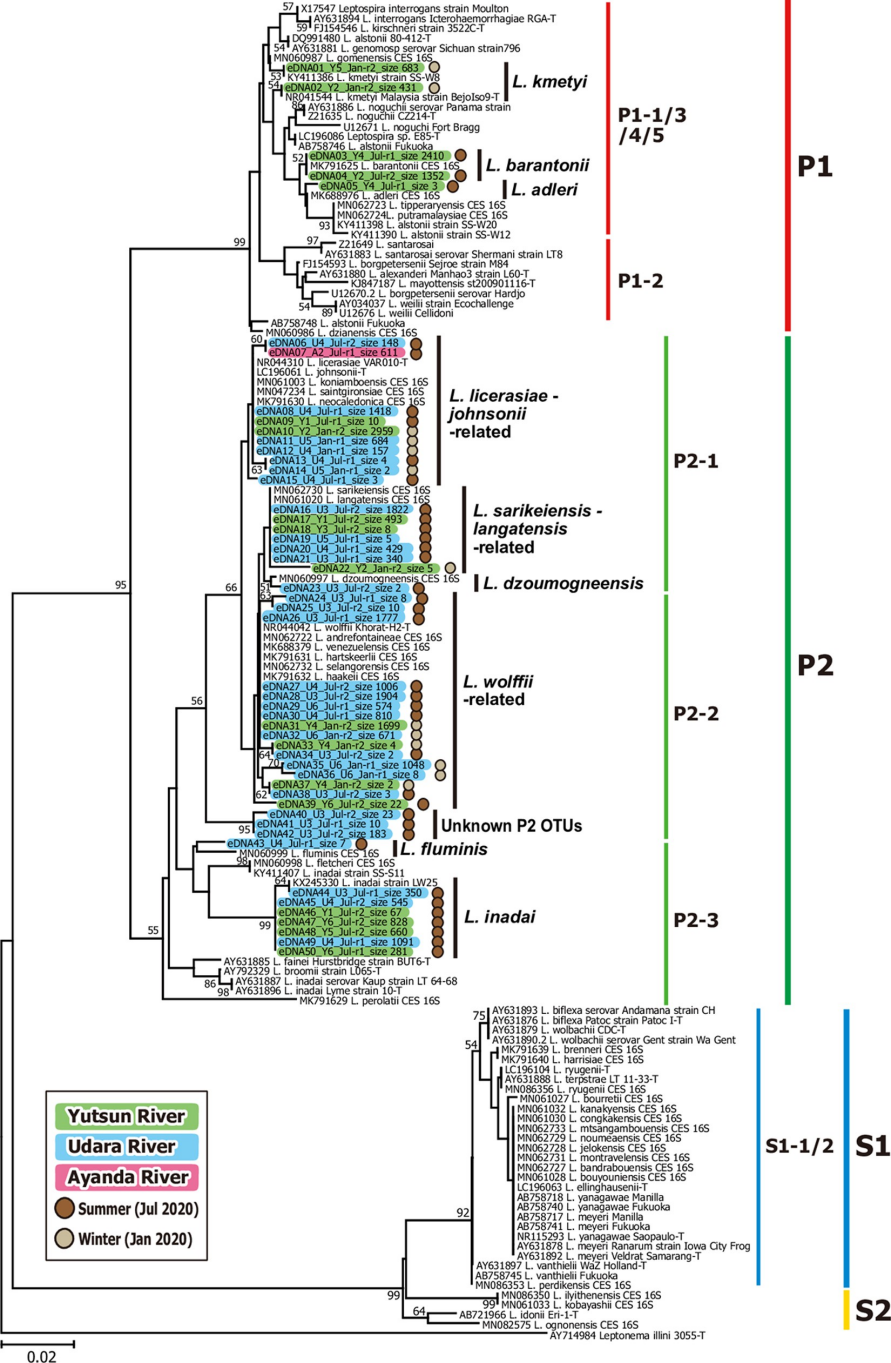
Molecular phylogenetic tree of partial leptospiral 16S rRNA sequences from river water eDNA samples with those of representative *Leptospira* species. Green, blue, and magenta shading indicate the partial 16S rRNA sequences determined from Yutsun, Udara, and Ayanda Rivers, respectively (ranging from 293 to 294 bp). Their locational origins (Y1−Y7, U1−U6, and A1 and A2), sampling months (Jan. or Jul.), PCR replication numbers (r1 and r2), and total sequence counts are denoted within the sequence names. The sequence counts are indicated after the word “size”. Brown and beige dots on the right side of the sequence names show that the sequence was determined from summer (July 2020) or winter (January 2020) samples, respectively. The GenBank accession numbers of known 16S rRNA sequences of representative *Leptospira* species were denoted in the sequence names. In total 280 nucleotide sites among these 136 sequences were aligned and analyzed by the neighbor-joining method with Kimura’s two parameter model of nucleotide substitution. Values on the tree nodes denote percentage support for the node estimated from 1,000 bootstrap replications. Subclade annotations P1, P2, S1 and S2 are based on Guglielmini *et al*. (2019) [1].

Molecular phylogenetic analysis was performed for the obtained eDNA-derived sequences of the pathogenic leptospiral 16S rRNA gene with those from known representative *Leptospira* species (Fig 4), indicating that eDNA sequences included 10 operational taxonomic units (OTUs) of *Leptospira*. The eDNA-derived *Leptospira* sequences (in total, 27,572 as described above) were comprised of a total of 50 unique sequences. According to the molecular phylogeny (Fig 4), the leptospiral eDNA included sequences genetically related to *L*. *kmetyi*, *L*. *barantonii*, and *L*. *adleri* from the P1 subclade, each found in winter or summer. The leptospiral OTUs of the P2 subclade were also found in these eDNA sequences, although their species assignment was relatively obscure due to shorter sequences of the eDNA-derived amplicons. These included OTUs of closer relatives of *L*. *licerasiae*, *L*. *johnsonii*, and others, *L*. *sarikeiensis* and *L*. *langatensis*, and *L*. *wolffii* (Fig 4). They were found in both the summer and winter samples. The eDNA sequences genetically related to *L*. *dzoumogneensis*, *L*. *fluminis*, and *L*. *inadai* were only found in summer samples with relatively reliable taxonomic resolution. In addition, an unknown, potentially newly found species or strain was found in summer samples, which was named as “Unknown P2 OTUs” and clustered in a basal position to the P2-2 subgroup (Fig 4). The combined analysis of 281 nucleotide sites from the 16S rRNA gene sequences derived from eDNA and soil isolates in the same phylogenetic tree gave similar results at the subclade level, but less resolution at the subgroup level (S1 Fig) than the independent analysis of 620 nucleotide sites from soil isolates (Fig 2). Thus, culture isolates classified as genetically related to *L*. *alstonii* in Fig 2 (YS-13, -17, -18, and -22), showed a monophyletic clade with *L*. *kmetyi* type strain in S1 Fig.

### Environmental DNA analysis of vertebrates focusing on tetrapod potentially correlated with *Leptospira*

To address the endemic fauna of Iriomote Island surrounding the wild *Leptospira* life cycle and potential reservoir animals of the leptospires, we amplified vertebrate mt-12S rRNA gene partial fragments from the same 30 eDNA samples using the MiFish primer (Table 1) [38]. This PCR was replicated twice for each sample and sequenced independently, generating in total 2,971,594 pairs of raw sequences with 49,527 ± 7,018 reads per replicate (ranging from 649 to 272,736; DRA012231). After quality-based filtering of the reads, a total of 2,550,128 quality-filtered sequences remained with 42,502 ± 6,962 sequences per replicate (ranging from 77 to 263,643), for which both primer ends of vertebrate mt-12S rRNA were found.

From the filtered 2,550,128 sequences of vertebrate mt-12S rRNA, a total of 2,458,125 non-singleton (≥2 counts) effective sequences were conferred species annotation after re-mapping analysis of singleton reads and blast-based analysis using a customized version of the MiFish pipeline [52] with tetrapod data from the NCBI nt database [50]. The average number of annotated vertebrate eDNA sequences was 40,969 ± 6,777 per replicate (ranging from 9 to 258,920), indicating 119 vertebrate species, including seven mammalian, three avians, and 109 teleost fish species. The full list of the species and their read counts are given in S2 Table. The remaining sequences were the true singletons in a replicate or sequences with no hits in the BLAST database (69,324 and 22,679 sequences, respectively).

**Fig 5:**
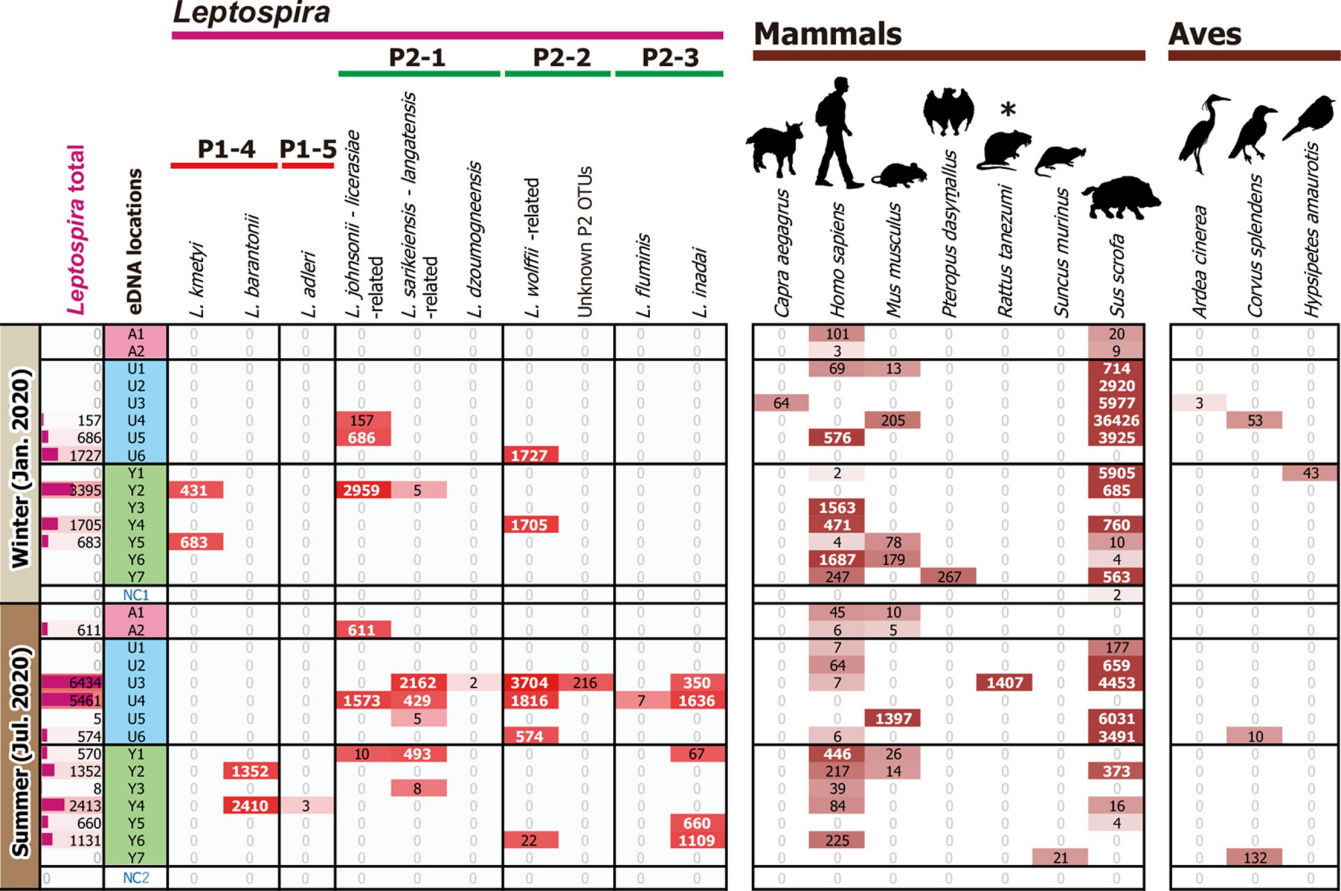
Environmental detection of leptospiral 16S rRNA gene and tetrapod mitochondrial 12S rRNA gene. Columns denote the species of *Leptospira* and tetrapods species, and rows denote the number of sequence reads detected in each sample. The reads are shown with colored matrices in red and brown shading for *Leptospira* and tetrapods, respectively, where the color intensity is relative to the read numbers. More than 300 reads are indicated by white bold white letters. P1-4 and -5, and P2-1 to -3 denote the phylogenetic subclades of *Leptospira* species based on Guglielmini *et al*. (2019) [1]. The leftmost “*Leptospira* total” column indicates the summed read numbers across all *Leptospira* species. Asterisks indicate tetrapod species that exhibited a significant correlation with detection of either species of *Leptospira* (Pearson’s product-moment correlation coefficient *r* > 0.361, *d*.*f*. = 28, two-tailed *p* < 0.05, and Benjamini–Hochberg-corrected false discovery rate *q* < 0.01). Y, U, and A indicate sampling locations Yutsun, Udara, and Ayanda Rivers, respectively. Y1−Y7, U1−U6, and A1 and A2 denote sample names. NC indicates negative control samples (RNase free water).

The correlation of eDNA detection between *Leptospira* and vertebrates focusing on tetrapods on Iriomote Island showed a significant relationship in rats (genus *Rattus*) (Fig 5, denoted by an asterisk). The sequence counts of each species from two PCR replicates (with different annealing temperatures; see Methods) were summed for each sample and are shown in Fig 5, since the detection pattern between the replicates was significantly correlated in both *Leptospira* and tetrapod (*r* = 0.424, *d*.*f*. = 30, *p* = 0.016 in the summed count “*Leptospira* total”; averaged *r* = 0.469, *d*.*f*. = 30, *p* = 0.007 across the 10 leptospiral OTUs; averaged *r* = 0.545, *d*.*f*. = 30, *p* = 0.001 across the 119 vertebrate species). The read numbers of “*Leptospira* total” were higher in summer than in winter, although the difference was not significant (1,281.27 ± 520.93 and 556.87 ± 255.66 reads in summer and winter, respectively; Welch’s *t* test, *d*.*f*. = 20.4, *t* = 0.96, *p* = 0.349). Shannon index of alfa-diversity of detected leptospiral OTUs was higher in summer (0.187 ± 0.104) than in winter (0.026 ± 0.026) although the difference was not significant (Welch’s *t* test, *d*.*f*. = 15.7 *t* = 1.50, *p* = 0.153). There was also no significant difference in averaged “*Leptospira* total” read numbers between rivers where the leptospirosis cases have been reported (Yutsun) or not (Udara and Ayanda) (851.21 ± 281.73 and 978.44 ± 500.34 reads in eastern Yutsun and western Udara and Ayanda, respectively; Welch’s *t* test, *d*.*f*. = 23.3, *t* = 0.22, *p* = 0.827). Shannon index of leptospiral alfa-diversity was higher in Udara (0.188 ± 0.128) than in Yutsun (0.068 ± 0.041) and Ayanda (0.000 ± 0.000) although that of Udara was not significantly different from Yutsun’s score (Welch’s *t* test, *d*.*f*. = 13.3, *t* = 0.89, *p* = 0.388). Among tetrapods, only rats significantly correlated with either *Leptospira* OTUs (*r* = 0.958 with *L*. *sarikeiensis*–*langatensis*-related, *r* = 1.000 with *L*. *dzoumogneensis* and unknown P2 OTUs, *r* = 0.766 with *L*. *wolffii*-related, and *r* = 0.649 with *Leptospira* total; *d*.*f*. = 28 [negative controls were excluded], *p* < 0.05, Benjamini–Hochberg-corrected FDR *q* < 0.01). The wild boar, a known host of *Leptospira* in Iriomote by serologic assays, was detected in most of the eDNA samples. However, there was no correlation with leptospiral detection (*r* was ranged from –0.107 to 0.057). When the more conservative, Fisher’s 2 x 2 exact test for the presence or absence of *Leptospira* or tetrapods was applied, a significant association was not detected for any tetrapod species including rats (two-tailed *p* = 0.433 to 1.000).

## Discussion

*Leptospira* species reported from clinical samples and animals in Yaeyama region belonged to the P1 subclade P1-1 (*L*. *interrogans* and *L*. *kirschneri*) and P1-2 (*L*. *borgpetersenii*) subgroups, including the serogroups Grippotyphosa, Hebdomadis, Icterohaemorrhagiae, Javanica, and Sejroe [3,15,56–58]. We first analyzed environmental isolates from soil collected along the Yutsun River, Iriomote Island, and successfully isolated several P1 subclade species genetically related to *L*. *kmetyi*, *L*. *barantonii* and *L*. *alstonii* that have not been previously reported in Yaeyama. Environmental soil isolates closely related to *L*. *alstonii and L*. *kmetyi* have been isolated from mainland Okinawa [19] and they have also been detected by eDNA metabarcoding in the northern part of Okinawa [29]. In contrast *L*. *barantonii* isolated from soil samples of New Caledonia [2,36] have not yet been reported in Japan. These species isolated from soil samples have been reported as probable novel pathogens, but could not induce signs or symptoms of infection in the hamster model, thus, their pathogenicity have not been proven [36]. We have not shown the presence of virulence related-genes by PCR, nor have proven that soil isolates induce cell-cell junction disruption as previously described for pathogenic *L*. *interrogans* [9]. Repeated *in vitro* subcultures attenuate the virulence of leptospires by drastically changing the protein expression profiles [10]. Thus, we could not rule out that during the isolation steps, which involved several passages in *in vitro*, the expression of bacterial factors involved in the disassembly of cell junctions was decreased. Interestingly, a clinical strain isolated in 2014 (strain 148233) might also have lost the ability to disrupt cell-cell junctions during long-term storage. *Leptospira kmetyi* was identified in a leptospirosis outbreak among canoeists in Martinique [59]; thus, *L*. *kmetyi* has been proven to be a pathogenic species that warrants attention in Yaeyama. To date, *L*. *alstonii* and *L*. *barantonii* have not been found in humans.

The analysis of eDNA metabarcoding extended to three rivers (Yutsun with greater access to tourists, and Udara and Ayanda, which are less accessible) during winter and summer, broaden our understanding of animal-environment-human relationships on Iriomote Island. Firstly, eDNA metabarcoding enabled the identification of OTUs corresponding to *Leptospira* species in the subgroups P2-1, P2-2 and P2-3 subgroups (*L*. *licerasiae*-related, *L*. *wolffii*-related, *L*. *inadai*, etc.) that were not detected by the bacterial culture protocol, even though they had less resolution at the subgroup level within each subclade, due to the shorter 16S rRNA sequences available for eDNA analysis than 16S PCR products obtained from soil isolates. Second, there was no significant difference in the strength of leptospiral eDNA detection between the eastern and western rivers, where leptospirosis cases have been reported (Yutsun) and not reported (Udara and Ayanda), suggesting that leptospires are widely distributed in Iriomote Island, and the number of reported cases is influenced by anthropogenic activities. Although P2 subclade species are rarely isolated from leptospirosis patients in Japan, they are increasingly reported in leptospirosis patients worldwide. For example, *L*. *wolffi* was isolated from the urine of a patient with suspected leptospirosis in Thailand [60] and *L*. *licerasiae* from the blood culture of a traveler returning to Japan from Brazil [61]. The *L*. *licerasiae* strain isolated from this traveler expresses LipL32, but does not express LigA or LigB [61], suggesting that some outer membrane proteins considered virulence-related are not essential for pathogenicity.

The subtropical climate of the Yaeyama region is favorable to a very diverse fauna with the potential to be *Leptospira* reservoir animals; continuous surveillance is, therefore, important for evidence-based control strategies for this zoonotic disease. A previous survey of leptospiral reservoir animals in Okinawa Prefecture showed that the wild boar (*Sus scrofa riukiuanus*) is a significant reservoir of *Leptospira* in Iriomote Island [30]. Although the direct detection of leptospires in the urine and/or kidneys of animals is the easiest approach to identifying reservoirs, it is sometimes difficult from a practical point of view in wild environments such as that in Yaeyama. Recently, the potential of using an eDNA/eRNA-based approach has been proposed as a powerful method for monitoring disease vectors/hosts in aquatic environments and understanding the spread of infectious diseases to evaluate human health risks in a given area [62]. This study applied a previously developed eDNA metabarcoding method to analyse the *Leptospira*–animals correlation and identify potential reservoir animals [29]. Our vertebrate eDNA analysis showed the presence of *S*. *scrofa* in almost all river samples, but there was no correlation with leptospiral detection. Interestingly, a higher number of *S*. *scrofa* read counts were obtained in winter than in summer. In Okinawa, the hunting season of them runs from November 15 to February 15; however, we don’t have additional evidences to show that the extremely high number read counts in U4/ winter reflects the *S*. *scrofa*-DNA derived from hunting-related activities. *Rattus* sp. is recognized worldwide as one of the main *Leptospira* reservoir animals [4,63]. We consistently found a significant correlation *r* between *Leptospira* and *Rattus* sp. (Fig 5) in the Udara river in summer. However, we could not find any correlation between animal-*Leptospira* in the Yutsun River in summer, where leptospirosis cases have been reported. The analysis of animals-*Leptospira* correlation using more conservative test based on the presence or absence of data showed no significant correlation suggesting that the conclusion of our data might be biased by the statistical analysis. Thus, further surveys and technical improvements in eDNA analysis are needed to clarify the animal reservoir around this river.

It should be noted that this study has several limitations as clinical and soil isolates and eDNA analyses were partially unrelated, which obscure some of our conclusions. Furthermore, our results using renal epithelial cells, suggested that experiments to show virulence should be performed with lowered passages of isolates, which is currently technically difficult using the conventional experimental protocols. The widespread detection of wild boar and human eDNA might impair the recognition of their correlation with leptospiral DNA. We consider that their widespread detection reflects the natural habitats and biomass and is not an artificial contamination. This is because the humans are more detected from Yutsun river (near the city) than other rivers; and boars detection is higher in Udara river (far remote from the city) than other sampling sites. In addition, there is nearly no detection from negative control samples. As a potential reservoir animal for *Leptospira*, we found a correlation of leptospiral eDNA with *Rattus* spp., however, *Rattus* spp. was identified in one sample out of 30, thus, a false correlation could not be rule out. In future work, we will strengthen the sample collection and experimental design to elucidate the environmental persistence and pathogenicity of *Leptospira* P1 species in leptospirosis-endemic regions.

## Supporting information

S1 Fig Molecular phylogenetic tree of partial leptospiral 16S rRNA sequences from river water eDNA and soil culture samples with those of representative *Leptospira* species. Green, blue, and magenta shading indicate the partial 16S rRNA sequences determined from Yutsun, Udara, and Ayanda rivers eDNA, respectively (ranging from 293 to 294 bp). The locational origins (Y1−Y7, U1−U6, and A1 and A2), sampling months (Jan. or Jul.), PCR replication numbers (r1 and r2), and total sequence counts are denoted within the sequence names. The sequence counts are indicated after the word “size”. Brown and beige dots on the right side of the sequence names show that the sequence was determined from summer (July 2020) or winter (January 2020) samples, respectively. Orange shading indicate the 16S rRNA sequences of *Leptospira* cultures from soil samples of the Yutsun riversides (ranging from 1,446 to 1,581 bp). Their GenBank accession numbers and those of representative *Leptospira* species were shown in the sequence names. In total 281 nucleotide sites among the 154 sequences were aligned and analyzed by the neighbor-joining method with Kimura’s two parameter model of nucleotide substitution. Values on the tree nodes denote percentage support for the node estimated from 1,000 bootstrap replications. Subclade annotations P1, P2, S1 and S2 are based on Guglielmini *et al*. (2019) [1].
(TIF)

S1 Table Concentration and quality of the extracted DNA from water samples. (DOCX)

S2 Table Sequence counts and taxonomic profiling of vertebrates detected from water samples. (XLSX)
