# Supplementary figures and images for: Analysis of human clinical and environmental Leptospira to elucidate the eco-epidemiology of leptospirosis in Yaeyama, subtropical Japan
Source: PLoS Negl Trop Dis. 2022 Mar 31;16(3):e0010234. doi: 10.1371/journal.pntd.0010234 (PMC8970387; doi:10.1371/journal.pntd.0010234)

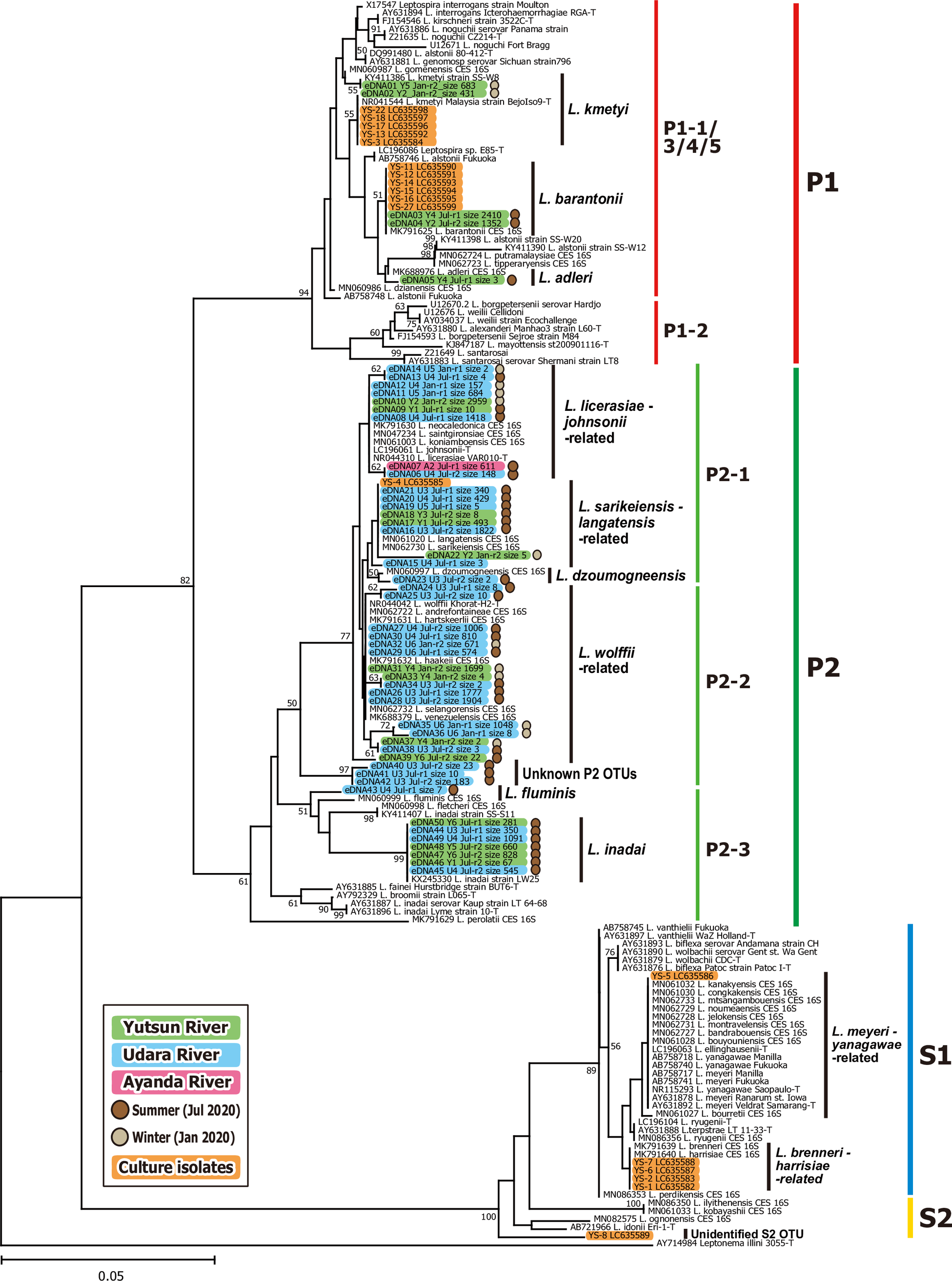

Supplement: S1 Fig — Green, blue, and magenta shading indicate the partial 16S rRNA sequences determined from Yutsun, Udara, and Ayanda rivers eDNA, respectively (ranging from 293 to 294 bp). The locational origins (Y1−Y7, U1−U6, and A1 and A2), sampling months (Jan. or Jul.), PCR replication numbers (r1 and r2), and total sequence counts are denoted within the sequence names. The sequence counts are indicated after the word “size”. Brown and beige dots on the right side of the sequence names show that the sequence was determined from summer (July 2020) or winter (January 2020) samples, respectively. Orange shading indicate the 16S rRNA sequences of Leptospira cultures from soil samples of the Yutsun riversides (ranging from 1,446 to 1,581 bp). Their GenBank accession numbers and those of representative Leptospira species were shown in the sequence names. In total 281 nucleotide sites among the 154 sequences were aligned and analyzed by the neighbor-joining method with Kimura’s two parameter model of nucleotide substitution. Values on the tree nodes denote percentage support for the node estimated from 1,000 bootstrap replications. Subclade annotations P1, P2, S1 and S2 are based on Guglielmini et al. (2019) [1]. (TIF) [file pntd.0010234.s001.tif]
