# Supplementary material for: Analysis of human clinical and environmental Leptospira to elucidate the eco-epidemiology of leptospirosis in Yaeyama, subtropical Japan
Source: PLoS Negl Trop Dis. 2022 Mar 31;16(3):e0010234. doi: 10.1371/journal.pntd.0010234 (PMC8970387; doi:10.1371/journal.pntd.0010234)
Supplement: S1 Table — (DOCX) [file pntd.0010234.s002.docx]

| **S1 Table. Concentration and quality of the extracted DNA.** | | | |  |  |
| --- | --- | --- | --- | --- | --- |
|  | Location name^a^ | Date of sampling | DNA concentration (ng/µl) | OD_260/280_ | Amounts of water filtered (mL) |
| Winter samples | |  |  |  |  |
|  | A1 | Jan. 10, 2020 | 2.10 | 2.24 | 600 |
|  | A2 | Jan. 10, 2020 | 1.70 | 4.48 | 800 |
|  | U1 | Jan. 10, 2020 | 1.40 | 4.45 | 600 |
|  | U2 | Jan. 10, 2020 | 2.50 | 2.33 | 1,000 |
|  | U3 | Jan. 10, 2020 | 1.10 | 2.55 | 1,000 |
|  | U4 | Jan. 10, 2020 | 1.70 | 2.76 | 950 |
|  | U5 | Jan. 10, 2020 | 1.20 | 6.48 | 1,000 |
|  | U6 | Jan. 10, 2020 | 1.30 | 7.18 | 1,000 |
|  | Y1 | Jan. 11, 2020 | 2.00 | 2.69 | 1,000 |
|  | Y2 | Jan. 11, 2020 | 0.90 | 2.03 | 1,000 |
|  | Y3 | Jan. 11, 2020 | 0.80 | -2.78 | 1,000 |
|  | Y4 | Jan. 11, 2020 | 0.80 | 1.90 | 1,000 |
|  | Y5 | Jan. 11, 2020 | 1.20 | 1.91 | 1,000 |
|  | Y6 | Jan. 11, 2020 | 1.00 | 2.17 | 1,000 |
|  | Y7 | Jan. 11, 2020 | 1.30 | 2.60 | 1,000 |
| Summer samples | |  |  |  |  |
|  | A1 | Jul. 12, 2020 | 7.30 | 2.59 | 800 |
|  | A2 | Jul. 12, 2020 | 2.90 | 3.29 | 800 |
|  | U1 | Jul. 12, 2020 | 2.30 | 5.42 | 800 |
|  | U2 | Jul. 12, 2020 | 2.10 | 2.49 | 700 |
|  | U3 | Jul. 12, 2020 | 1.10 | -2.09 | 800 |
|  | U4 | Jul. 12, 2020 | 1.10 | -1.34 | 800 |
|  | U5 | Jul. 12, 2020 | 1.20 | -2.83 | 1,000 |
|  | U6 | Jul. 12, 2020 | 1.90 | 8.85 | 1,000 |
|  | Y1 | Jul. 10, 2020 | 3.70 | 2.48 | 900 |
|  | Y2 | Jul. 10, 2020 | 1.80 | 5.36 | 1,000 |
|  | Y3 | Jul. 10, 2020 | 1.50 | 5.38 | 1,000 |
|  | Y4 | Jul. 10, 2020 | 1.50 | 14.82 | 1,000 |
|  | Y5 | Jul. 10, 2020 | 2.60 | 3.58 | 1,000 |
|  | Y6 | Jul. 10, 2020 | 2.60 | 2.65 | 1,000 |
|  | Y7 | Jul. 10, 2020 | 1.70 | 2.68 | 1,000 |
| ^a^Detailed locations of the sampling sites are indicated in Fig. 1. | | | |  |  |
